# Loss of stearoyl-CoA desaturase 2 disrupts inflammatory response in macrophages

**DOI:** 10.1128/mbio.00925-23

**Published:** 2023-07-07

**Authors:** Joseph B. Lin, Amy Mora, Tzu Jui Wang, Andrea Santeford, Darksha Usmani, Marianne M. Ligon, Indira U. Mysorekar, Rajendra S. Apte

**Affiliations:** 1 John F. Hardesty, MD Department of Ophthalmology and Visual Sciences, Washington University School of Medicine, St. Louis, Missouri, USA; 2 Neurosciences Graduate Program, Roy and Diana Vagelos Division of Biology & Biomedical Sciences, Washington University School of Medicine, St. Louis, Missouri, USA; 3 Department of Obstetrics & Gynecology, Washington University School of Medicine, St. Louis, Missouri, USA; 4 Department of Medicine, Section of Infectious Diseases, Baylor College of Medicine, Houston, Texas, USA; 5 Department of Molecular Virology and Microbiology, Baylor College of Medicine, Houston, Texas, USA; 6 Department of Developmental Biology, Washington University School of Medicine, St. Louis, Missouri, USA; 7 Center of Regenerative Medicine, Washington University School of Medicine, St. Louis, Missouri, USA; 8 Department of Medicine, Washington University School of Medicine, St. Louis, Missouri, USA; University of Kentucky, Lexington, Kentucky, USA

**Keywords:** macrophage, UPEC, IL1B, inflammaging, cardiolipin, stearoyl-CoA desaturase

## Abstract

Macrophages are innate immune cells that patrol tissues and are the first responders to detect infection. They orchestrate the host immune response in eliminating invading pathogens and the subsequent transition from inflammation to tissue repair. Macrophage dysfunction contributes to age-related pathologies, including low-grade inflammation in advanced age that is termed “inflammaging.” Our laboratory has previously identified that macrophage expression of a fatty acid desaturase, stearoyl-CoA desaturase 2 (SCD2), declines with age. Herein, we delineate the precise cellular effects of SCD2 deficiency in murine macrophages. We found that deletion of *Scd2* from macrophages dysregulated basal and bacterial lipopolysaccharide (LPS)-stimulated transcription of numerous inflammation-associated genes. Specifically, deletion of *Scd2* from macrophages decreased basal and LPS-induced expression of *Il1b* transcript that corresponded to decreased production of precursor IL1B protein and release of mature IL1B. Furthermore, we identified disruptions in autophagy and depletion of unsaturated cardiolipins in SCD2-deficient macrophages. To assess the functional relevance of SCD2 in the macrophage response to infection, we challenged SCD2-deficient macrophages with uropathogenic *Escherichia coli* and found that there was impaired clearance of intracellular bacteria. This increased burden of intracellular bacteria was accompanied by increased release of pro-inflammatory cytokines IL6 and TNF but decreased IL1B. Taken together, these results indicate that macrophage expression of *Scd2* is necessary for maintaining the macrophage response to inflammatory stimuli. This link between fatty acid metabolism and fundamental macrophage effector functions may potentially be relevant to diverse age-related pathologies.

**IMPORTANCE:**

Macrophages are immune cells that respond to infection, but their dysfunction is implicated in many age-related diseases. Recent evidence showed that macrophage expression of a fatty acid enzyme, stearoyl-CoA desaturase 2, declines in aged organisms. In this work, we characterize the effects when stearoyl-CoA desaturase 2 is deficient in macrophages. We identify aspects of the macrophage inflammatory response to infection that may be affected when expression of a key fatty acid enzyme is decreased, and these findings may provide cellular insight into how macrophages contribute to age-related diseases.

## INTRODUCTION

Macrophages are the sentinel cells of the innate immune system that patrol nearly all tissues for pathogens (1). In the early stage of response, macrophages phagocytose microbes, process and present foreign antigens to recruit the adaptive immune response, and also manage the inflammatory response by releasing pro-inflammatory cytokines (1). In later stages, macrophages also mediate the transition from inflammation that eliminates invading pathogens to tissue repair and wound healing (1). In addition to their critical role in the host response to infection, macrophages have been shown to contribute to inflammation that occurs with aging (2) and may have widespread contributions to cardiovascular disease, pathologies of the genitourinary system (3), eye diseases (4), and cancers (5).

In our previous studies, we have demonstrated that macrophage dysfunction in aging is coordinated in part by perturbations in lipid homeostasis (6 - 8). Specifically, we have identified age-associated downregulation of stearoyl-CoA desaturase 2 (SCD2) (7), an enzyme that converts the long-chain fatty acids 16:0 and 18:0 to the monounsaturated fatty acids 16:1 and 18:1, respectively. Several recent studies have shown that SCD fatty acid desaturation regulates macrophage-mediated inflammation (9, 10). Macrophage activation leads to altered abundance of monounsaturated fatty acids including those produced by SCD2, and deletion of SCD1/2 prolonged upregulation of cytokines elicited by inflammatory stimuli (9). In the demyelinating disease multiple sclerosis, microglia (i.e., resident macrophages of the central nervous system) upregulate SCD1 in response to accumulation of phagocytosed myelin, which ultimately increased inflammation preventing resolution of lesions (10). Our laboratory has shown that macrophage-specific deletion of *Scd2* increases pathologic angiogenesis in the eye in a mouse model of the blinding disease age-related macular degeneration (7).

However, a cell-intrinsic, functional role for SCD2 in macrophages during the inflammatory response to infection has not been explored. In this work, we investigated the role of *Scd2* in macrophages by studying *Scd2-*deficient macrophages deleted via lysozyme M-Cre recombinase. We show that SCD2-deficient macrophages exhibit dysregulation of several inflammatory genes at baseline and in response to lipopolysaccharide (LPS) stimulation. Though SCD2 was not required for general NFkB activation, it was necessary for IL1B activation following LPS stimulation. Specifically, we identified that there was a deficit in signal 1 that is responsible for induction of *Il1b* mRNA that is translated to precursor IL1B, whereas SCD2 was not necessary for signal 2 that cleaves pro-IL1B and releases it. Furthermore, we found that there were disruptions to autophagy as well as dramatically decreased amounts of unsaturated cardiolipins (CL). Finally, we tested the relevance of these findings to the macrophage response to microbes by challenging SCD2-deficient macrophages with a uropathogenic strain of *Escherichia coli* (UPEC). Loss of SCD2 was associated with impaired clearance of intracellular UPEC as well as a concomitant increase in the release of IL6 and TNF but decreased IL1B release. Therefore, we identify a novel role for SCD2-mediated fatty acid desaturation in macrophage function during infection response.

## RESULTS

### SCD2 deficiency dysregulates inflammatory gene expression in macrophages

**Fig 1:**
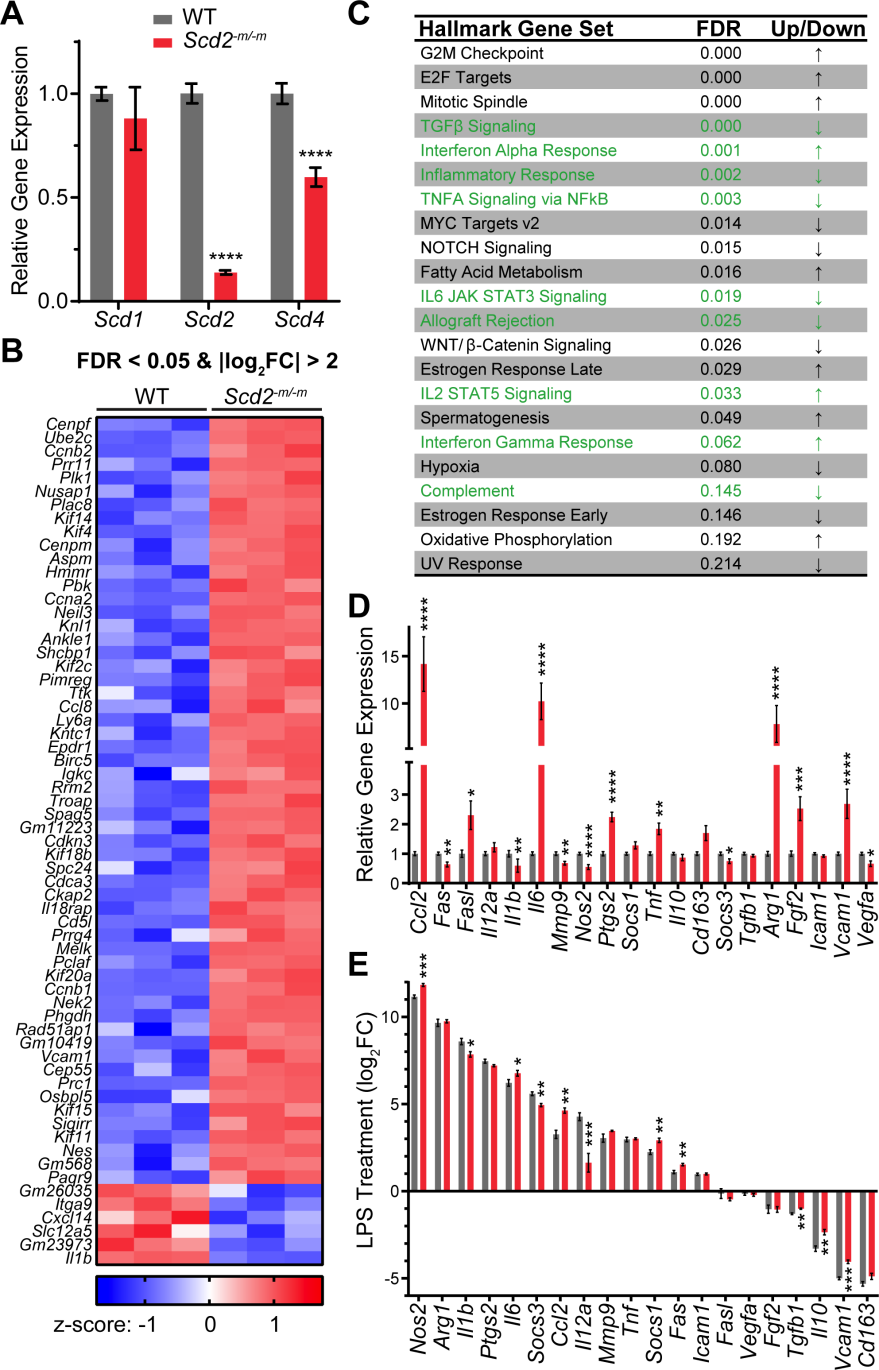
Deficiency of SCD2 perturbs macrophage inflammatory gene expression and response. (**A**) Bar graphs showing gene expression of *Scd1, Scd2,* and *Scd4* in wild-type (WT) macrophages and macrophages with lysozyme M-Cre-driven deletion of *Scd2* (*Scd2^-m/-m^*). *Scd1/2/4* gene expression was normalized to expression of housekeeping genes *Stx5a* and *Hnrnpab*. Each bar shows the mean ± SEM for *n* = 12–16 wells from three to four independent experiments. Statistical significance was assessed using Mann-Whitney tests. (**B**) Heat map showing the top differentially expressed genes in *Scd2^-m/-m^* macrophages. Genes with false discovery rate (FDR) <0.05 and |log_2_FC| >2 are shown. (**C**) Table of pathways identified to be dysregulated in *Scd2^-m/-m^* macrophages. (**D**) Profiling of inflammatory genes in *Scd2^-m/-m^* macrophages. Gene expression was normalized to expression of *18S, Actb, Gapdh,* and *Hprt*. Each bar indicates the mean ± SEM of *n* = 12 wells from two independent experiments. Statistical significance was assessed using Mann-Whitney tests. (**E**) Profiling of inflammatory gene response in *Scd2^-m/-m^* macrophages after LPS treatment (100 ng/mL, 24 h). Gene expression was normalized to expression of *18S, Actb, Gapdh,* and *Hprt* and then expressed as the log_2_ of the fold change relative to untreated cells. Each bar indicates the mean ± SEM of *n* = 7–8 wells from two independent experiments. Statistical significance was assessed using the Mann-Whitney test. Statistical significance is indicated with asterisks as follows: *, *P* < 0.05; **, *P* < 0.01; ***, *P* < 0.001; ****, *P* < 0.0001.

In order to first confirm *Scd2* deletion in macrophages, we prepared bone marrow-derived macrophages (BMDMs) from wild-type mice and from mice with conditional deletion of *Scd2* in myeloid cells driven by the lysozyme M-Cre recombinase (*Scd2^-m/-m^*). We found that in macrophages prepared from *Scd2^-m/-m^* mice, *Scd2* transcript was ~20% that of macrophages prepared from wild-type mice (Fig. 1A). We also measured expression of *Scd1* and *Scd4,* the other two SCD isoforms with detectable expression in macrophages. There was no compensatory upregulation by either *Scd1* or *Scd4* in *Scd2^-m/-m^* macrophages, though *Scd4* was downregulated by 40% (Fig. 1A). It has been previously reported that *Scd2* is expressed at a much higher level than *Scd4* in BMDMs with the latter not being detected in RNA sequencing data (9). Together, these data indicate that *Scd2* deletion from macrophages does not lead to compensatory upregulation of other SCD isoforms at the mRNA level.

To broadly assess the transcriptional changes that occur upon deletion of *Scd2*, we performed RNA sequencing. We obtained an average of 35.4 million reads per samples (*n* = 3 wild type, *n* = 3 *Scd2^-m/-m^*). Of these, 35.2 million reads (99.6%) were mapped to the mouse genome. There were 63 differentially expressed genes with false discovery rate (FDR) <0.05 and |log_2_FC| >2 (Fig. 1B). To determine whether or not these transcriptional changes could be indicative of specific cellular pathways, we performed pathway analysis using Gene Set Enrichment Analysis using the hallmark gene sets database (11, 12). There were 22 pathways with FDR below the recommended cutoff of 0.25 (Fig. 1C). Ten pathways were upregulated in *Scd2^-m/-m^* macrophages and the remaining 12 were downregulated. There was mixed dysregulation of pro-inflammatory and anti-inflammatory pathways, including transforming growth factor beta (TGFB) signaling, interferon alpha response, inflammatory response, TNFA signaling via NFkB, IL6-JAK-STAT3 signaling, allograft rejection, IL2-STAT5 signaling, interferon gamma response, and complement (Fig. 1C). Notably, pathway analysis also identified fatty acid metabolism as a perturbed pathway in SCD2-deficient macrophages (Fig. 1C), consistent with SCD2’s role as a fatty acid desaturase.

We then compared mRNA expression of 20 macrophage activation markers. In naïve macrophages, deletion of *Scd2* caused dysregulation of 14 of the 20 genes assessed (Fig. 1D). Of these, eight were upregulated (*Ccl2, Fasl, Il6, Ptgs2, Tnf, Arg1, Fgf2,* and *Vcam1*), and six were downregulated (*Fas, Il1b, Mmp9, Nos2, Socs3,* and *Vegfa*). Some pro-inflammatory genes were upregulated (e.g., *Il6* and *Tnf*), whereas others were downregulated (e.g., *Il1b*). Similarly, some anti-inflammatory markers were upregulated (e.g., *Arg1*), whereas others were downregulated (e.g., *Vegfa*). To assess macrophage response to inflammatory stimuli, we also compared the fold change of these 20 genes after activation of macrophages with the Gram-negative bacterial membrane component LPS (100 ng/mL) for 24 h. All genes assessed were responsive to LPS with the same direction of change in both wild-type and *Scd2^-m/-m^* macrophages (Fig. 1E). However, there were six genes with blunted responses to LPS (*Il1b, Socs3, Il12a, Tgfb1, Il10,* and *Vcam1*) and five genes with an exaggerated response to LPS (*Nos2, Il6, Ccl2, Socs1,* and *Fas*). Taken together, these gene expression data suggest that the deletion of *Scd2* from macrophages perturbs inflammatory gene expression at baseline and in response to inflammatory stimuli.

### SCD2 is dispensable for LPS-induced NFkB phosphorylation and nuclear translocation

**Fig 2:**
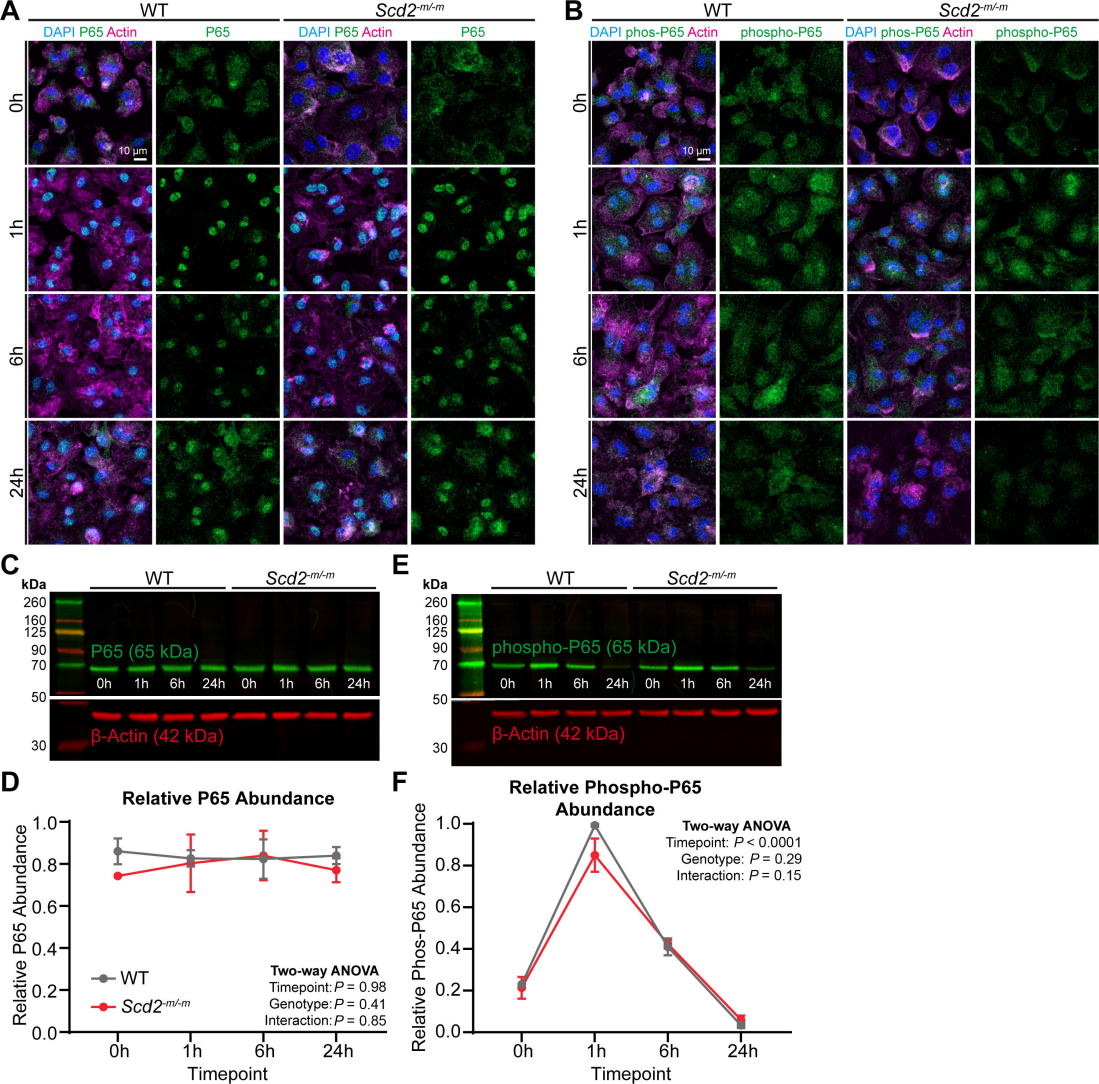
SCD2 is dispensable for NFkB activation. (**A**) Immunostaining for total P65 in *Scd2^-m/-m^* macrophages after LPS treatment (100 ng/mL). (**B**) Immunostaining for phosphorylated P65 in *Scd2^-m/-m^* macrophages after LPS treatment (100 ng/mL). (**C**) Representative immunoblot for total P65 in *Scd2^-m/-m^* macrophages after LPS treatment (100 ng/mL). (**D**) Line graph showing quantifications of total P65 in *Scd2^-m/-m^* macrophages after LPS treatment (100 ng/mL) from immunoblotting experiments. P65 band intensity was normalized to loading control β-actin band intensity, then normalized to the maximum lane’s value on each gel. Each point indicates the mean ± SEM for *n* = 3 samples from three independent experiments. Statistical significance was assessed using two-way analysis of variance (ANOVA) with Šídák’s multiple comparisons test. (**E**) Representative immunoblot for phospho-P65 in *Scd2^-m/-m^* macrophages after LPS treatment (100 ng/mL). (**F**) Line graph showing quantifications of phospho-P65 in *Scd2^-m/-m^* macrophages after LPS treatment (100 ng/mL) from immunoblotting experiments. Phospho-P65 band intensity was normalized to loading control β-actin band intensity, then normalized to the maximum lane’s value on the gel. Each point indicates the mean ± SEM for *n* = 3 samples from three independent experiments. Statistical significance was assessed using two-way ANOVA with Šídák’s multiple comparisons test. WT, wild type.

Because inflammatory stimuli like LPS are known to activate macrophages via NFkB signaling, we assessed for LPS-elicited phosphorylation and nuclear translocation of the NFkB subunit P65. After LPS treatment, there was substantial translocation of P65 from the cytoplasm into the nucleus that peaked at 1 h (Fig. 2A), along with a concomitant and slight increase in P65 phosphorylation (Fig. 2B). To quantify these changes, we performed western blot of whole macrophage lysates. Total cellular P65 abundance was stable up to 24 h after LPS treatment (Fig. 2C and D). P65 phosphorylation peaked at 1 h after LPS stimulation and decreased thereafter and was similar between wild-type and SCD2-deficient macrophages (Fig. 2E and F). These data indicate that SCD2 is dispensable for NFkB activation.

### SCD2 deletion from macrophages impairs IL1B activation

**Fig 3:**
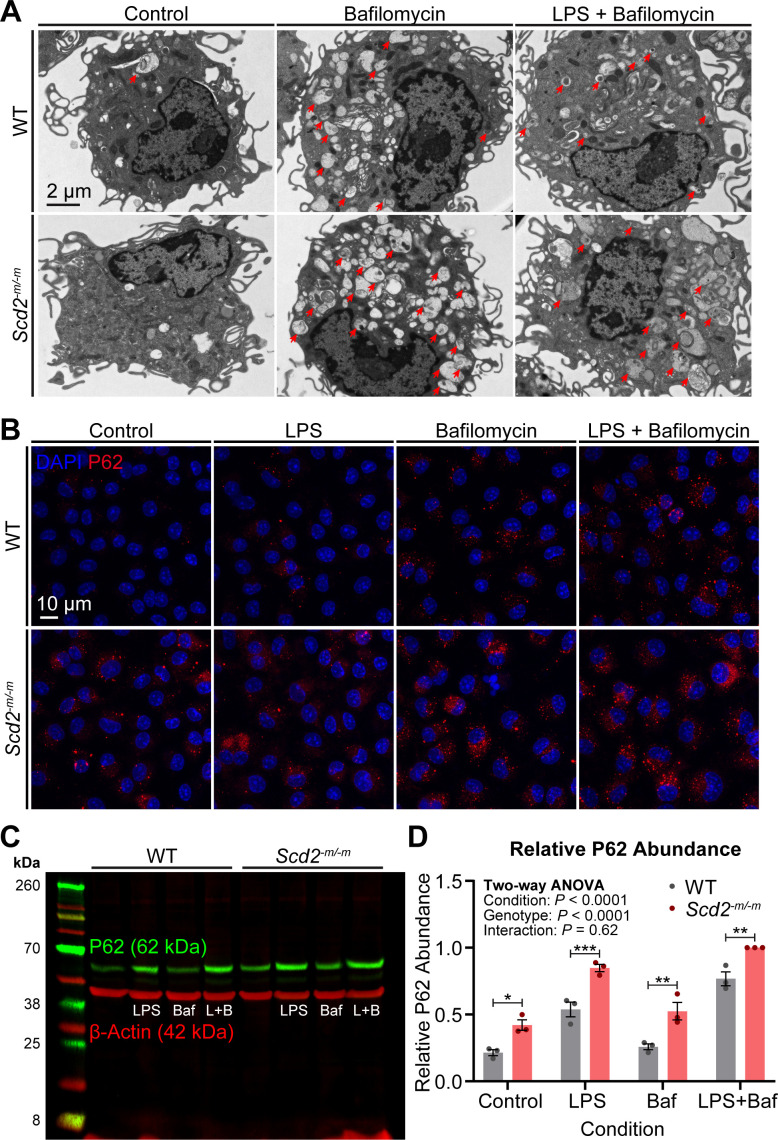
Loss of SCD2 disrupts autophagy in macrophages. (**A**) Transmission electron microscopy images showing increased accumulation of autophagosomes in *Scd2^-m/-m^* macrophages treated with bafilomycin A1 (800 nM) and/or LPS (100 ng/mL) for 2 h. Red arrows indicate double membrane-enclosed autophagosomes. (**B**) Immunostaining for the autophagosome marker P62. Macrophages were treated with bafilomycin A1 (800 nM) and/or LPS (100 ng/mL) for 2 h. (**C**) Representative immunoblot for P62 in macrophages treated with bafilomycin A1 (800 nM) and/or LPS (100 ng/mL) for 2 h. (**D**) Bar graph showing quantifications of P62 (62 kDa) from immunoblotting experiments. P62 band intensity was normalized to loading control β-actin band intensity, then normalized to the maximum lane’s value on the gel. Each bar indicates the mean ± SEM for *n* = 3 samples from three independent experiments. Statistical significance was assessed using two-way analysis of variance (ANOVA) with Šídák’s multiple comparisons test and is indicated with asterisks as follows: *, *P* < 0.05; **, *P* < 0.01; ***, *P* < 0.001. WT, wild type.

It is known that inflammation is regulated by autophagy (13, 14). We found there was general dysregulation of pro-inflammatory markers *Il6, Tnf,* and *Il1b* in *Scd2^-m/-m^* macrophages (Fig. 1D). As such, we tested whether there were any changes in autophagic clearance. In transmission electron micrographs, we noted that there appeared to be increased accumulation of autophagosomes as identified by cytoplasmic double-membraned vesicles (Fig. 3A). Next, we stained for the autophagy substrate marker, P62, and, consistent with electron microscopic evaluation, there was increased P62 abundance in *Scd2^-m/-m^* macrophages under all conditions tested (Fig. 3B). To quantify these changes, we immunoblotted for P62 (Fig. 3C and D). There was a statistically significant increase in P62 abundance in *Scd2^-m/-m^* macrophages (Fig. 3D). Therefore, loss of SCD2 appears to disrupt autophagic flux in macrophages via P62.

**Fig 4:**
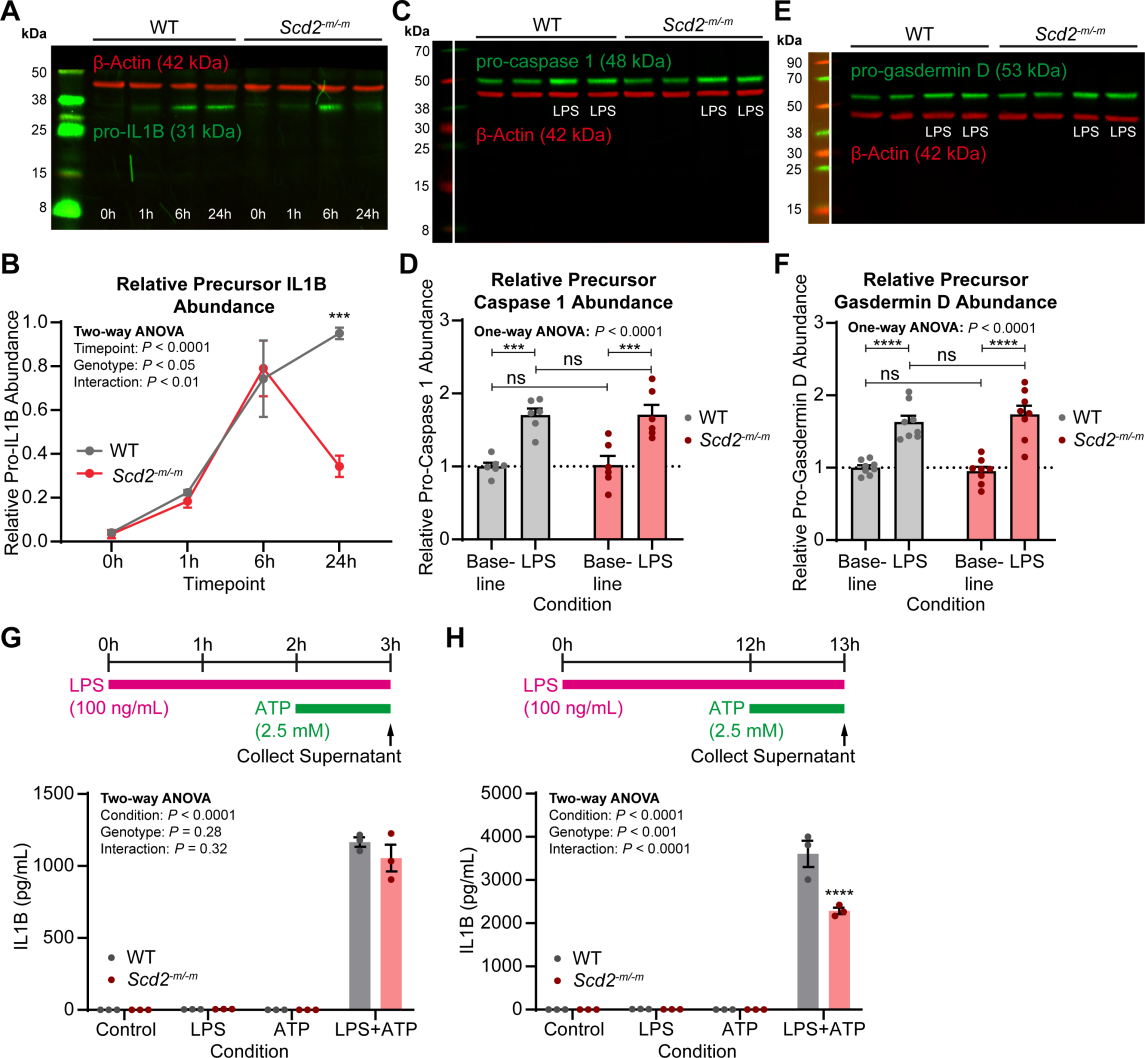
SCD2 is required for IL1B activation and release. (**A**) Representative immunoblot for IL1B in *Scd2^-m/-m^* macrophages after LPS treatment (100 ng/mL). For aesthetic purposes only, the blot was placed on a black background such that the image is surrounded evenly by dark margins. (**B**) Line graph showing quantifications of precursor IL1B (31 kDa) in *Scd2^-m/-m^* macrophages after LPS treatment (100 ng/mL) from immunoblotting experiments. Pro-IL1B band intensity was normalized to loading control β-actin band intensity, then normalized to the maximum lane’s value on the gel. Each point indicates the mean ± SEM for *n* = 3 samples from three independent experiments. Statistical significance was assessed using two-way analysis of variance (ANOVA) with Šídák’s multiple comparisons test. (**C**) Representative immunoblot for caspase 1 in *Scd2^-m/-m^* macrophages after LPS treatment (100 ng/mL, 24 h). For enhanced clarity, the left-most molecular weight marker lane is comprised of two images of the ladder with two separate brightness and contrast settings applied that have been joined together at the indicated splice line. (**D**) Bar graph showing quantifications of precursor caspase 1 (48 kDa) in *Scd2^-m/-m^* macrophages after LPS treatment (100 ng/mL, 24 h) from immunoblotting experiments. Pro-caspase 1 band intensity was normalized to loading control β-actin band intensity, then normalized to the average value for untreated wild-type (WT) samples in each experiment. Each point indicates the mean ± SEM for *n* = 6 samples from three independent experiments. Statistical significance was assessed using one-way ANOVA with Šídák’s multiple comparisons test. (**E**) Representative immunoblot for gasdermin D in *Scd2^-m/-m^* macrophages after LPS treatment (100 ng/mL, 24 h). For enhanced clarity, the left-most molecular weight marker lane is comprised of two images of the ladder with two separate brightness and contrast settings applied that have been joined together at the indicated splice line. (**F**) Bar graph showing quantifications of precursor gasdermin D (53 kDa) in *Scd2^-m/-m^* macrophages after LPS treatment (100 ng/mL, 24 h) from immunoblotting experiments. Pro-gasdermin D band intensity was normalized to loading control β-actin band intensity, then normalized to the average value for untreated WT samples in each experiment. Each point indicates the mean ± SEM for *n* = 8 samples from four independent experiments. Statistical significance was assessed using one-way ANOVA with Šídák’s multiple comparisons test. (**G–H**) Bar graphs showing quantifications of released IL1B in cells treated sequentially with LPS (100 ng/mL) then adenosine triphosphate (ATP) (2.5 mM). Each bar indicates the mean ± SEM for *n* = 3 wells/group. Statistical significance was assessed using two-way ANOVA with Šídák’s multiple comparisons test. Statistical significance is indicated with asterisks as follows: ***, *P* < 0.001; ****, *P* < 0.0001.

Altered autophagic flux can lead to decreased IL1B signaling by affecting P62 degradation (15 - 17); thus, we next examined whether IL1B secretion was affected in *Scd2^-m/-m^* macrophages. IL1B is a pro-inflammatory cytokine whose release by macrophages is activated by two sequential signals. After signal 1, *Il1b* is transcribed and translated into pro-IL1B protein (31 kDa). After signal 2, the precursor IL1B protein is cleaved into mature IL1B protein (17 kDa) by caspase 1 and then released nonspecifically through gasdermin pores during pyroptosis. LPS activates the first signal via NFkB. Though NFkB nuclear translocation and phosphorylation were unaffected in SCD2-deficient macrophages, *Il1b* mRNA was downregulated at baseline (Fig. 1B and D) and its activation was blunted after LPS stimulation (Fig. 1E). Therefore, we assessed IL1B protein by western blotting and found that in SCD2-deficient macrophages, there was appropriate induction of pro-IL1B up until 6 h after LPS stimulation (Fig. 4A and B). However, at the later 24-h timepoint, there was significantly less pro-IL1B protein (Fig. 4A and B).

It is possible that decreased amounts of pro-IL1B are due to decreased translation or increased cleavage and/or release because of inappropriate constitutive signal 2 activity in SCD2-deficient macrophages. There was very little, if any, mature IL1B detected (Fig. 4A), suggesting that pro-IL1B is not constitutively cleaved. Nonetheless, we assessed for pro-caspase 1 and active caspase 1 (48 and 10 kDa, respectively), the latter of which cleaves pro-IL1B to mature IL1B. Consistent with lack of mature IL1B, there was very little, if any, active caspase 1 under the conditions tested (Fig. 4C). Baseline levels of pro-caspase 1 were similar in both wild-type and SCD2-deficient macrophages, and LPS treatment for 24 h induced a similar 1.7-fold increase in pro-caspase 1 for both genotypes (Fig. 4C and D). Finally, we assessed for precursor and cleaved gasdermin D (53 and 30 kDa, respectively), the latter of which forms pores on the plasma membrane that ultimately leads to pyroptosis and IL1B release. There was very little, if any, cleaved gasdermin D detected (Fig. 4E). Baseline levels of precursor gasdermin D were similar in both wild-type and SCD2-deficient macrophages, and LPS treatment induced a similar 1.6- to 1.7-fold increase in precursor gasdermin D for both genotypes (Fig. 4E and F).

To assess whether deficient induction of pro-IL1B leads to decreased release of mature IL1B to the extracellular space, we first activated signal 1 with LPS (100 ng/mL) followed by activation of signal 2 with ATP (2.5 mM). We performed this experiment with short (2 h) or long (12 h) LPS stimulation, both followed by the same 1 h of ATP stimulation (Fig. 4G and H). We collected media and measured released IL1B. Consistent with immunoblotting experiments, there was no difference in IL1B release from *Scd2^-m/-m^* macrophages that were stimulated acutely with LPS for 2 h (Fig. 4G). However, when *Scd2^-m/-m^* macrophages were stimulated with LPS for longer (12 h), there was a statistically significant decrease in the amount of IL1B released by SCD2-deficient macrophages (Fig. 4H). Therefore, deletion of SCD2 from macrophages impairs activation and release of IL1B, especially in the later phase of inflammatory activation. The delay in IL1B reduction after autophagy disruption may represent the time lag necessary for *Il1b* transcription and translation to protein and its cleavage, cytoplasmic transport, and extracellular release.

### Loss of macrophage SCD2 depletes unsaturated cardiolipins

**Fig 5:**
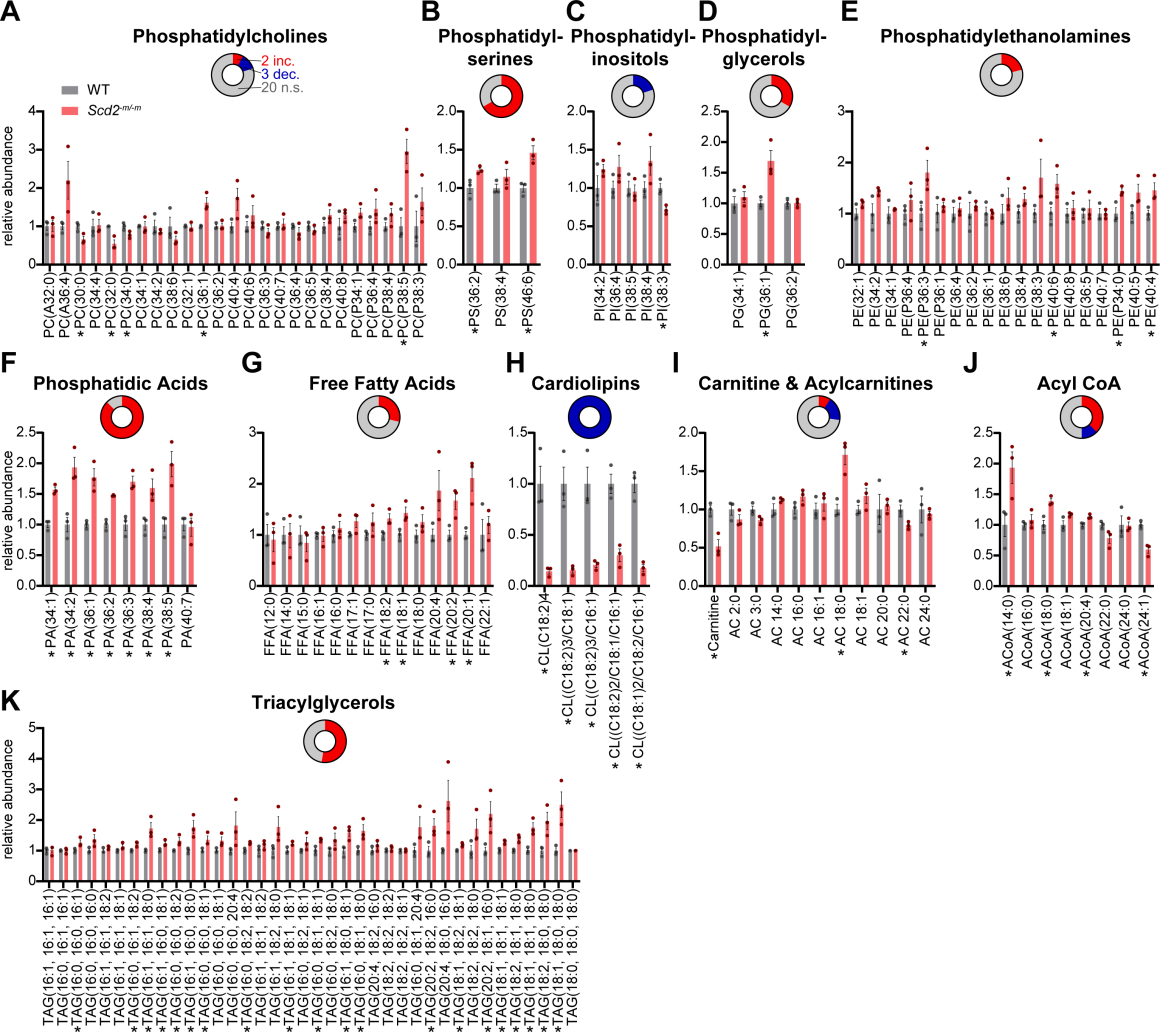
Effect of SCD2 deficiency on macrophage lipidome. Bar graphs showing quantifications of the following lipids in *Scd2^-m/-m^* macrophages: PC (**A**), PS (**B**), PI (**C**), PG (**D**), PE (**E**), PA (**F**), FFA (**G**), CL (**H**), carnitine and AC (**I**), ACoA (**J**), and TAG (**K**). Lipids were quantified relative to an internal standard and then normalized to the average ratios for wild-type (WT) samples. Each bar indicates the mean ± SEM for *n* = 3 samples/group. Statistical significance was assessed by calculating a two-sided *P*-value on the difference of means between two samples created by bootstrapping and is indicated by asterisks as follows: *, *P* < 0.05. Inset donut graph shows the proportion of species within each lipid class that are increased (red), decreased (blue), or unchanged (gray).

Because SCD2 is a fatty acid desaturase, we tested whether its deletion affected its substrates or products or other lipid species. Using liquid chromatography-mass spectrometry, we measured 11 classes of lipids: phosphatidylcholine (PC), phosphatidylserine (PS), phosphatidylinositol (PI), phosphatidylglycerol (PG), phosphatidylethanolamine (PE), phosphatidic acid (PA), free fatty acids (FFA), CL, carnitine and acylcarnitines (AC), acyl CoAs (ACoA), and triacylglycerols (TAG). Of the 139 lipid species assessed, 56 had statistically significant changes between wild-type and *Scd2^-m/-m^* macrophages with fold changes ranging from 0.14× to 2.96× (Fig. 5). For 7 of the 11 lipid classes that we assessed, there were mild changes with statistically significant differences in 1/2 or less of the lipid species detected within each class (Fig. 5A through K). For example, among the 25 phosphatidylcholine species assessed, there were increases in PC(P38:5) and PC(36:1), whereas PC(32:0), PC(30:0), and PC(34:0) were decreased; the remaining 20 phosphatidylcholines were unchanged. The other six classes with only mild changes were: PI, PG, PE, FFA, carnitines and AC, and ACoA. On the other hand, PS, PA, CL, and TAG had statistically significant differences in >1/2 of the individual species within each class. Individual species of PS, PA, and TAG with statistically significant changes were all increased in abundance, and all of these species contained monounsaturated or polyunsaturated acyl chains (Fig. 5B, F and K). On the other hand, all five unsaturated cardiolipins detected were decreased 3.3- to 7.1-fold compared to wild-type macrophages and were the only lipid species with more than threefold change out of the 139 lipid species assessed (Fig. 5H). Notably, cardiolipins have previously been shown to play a direct role in the priming and activation of the nucleotide-binding oligomerization domain-like receptors family pyrin domain containing 3 (NLRP3) inflammasome responsible for IL1B release (18, 19).

### SCD2 deficiency perturbs macrophage response to uropathogenic *E. coli*

**Fig 6:**
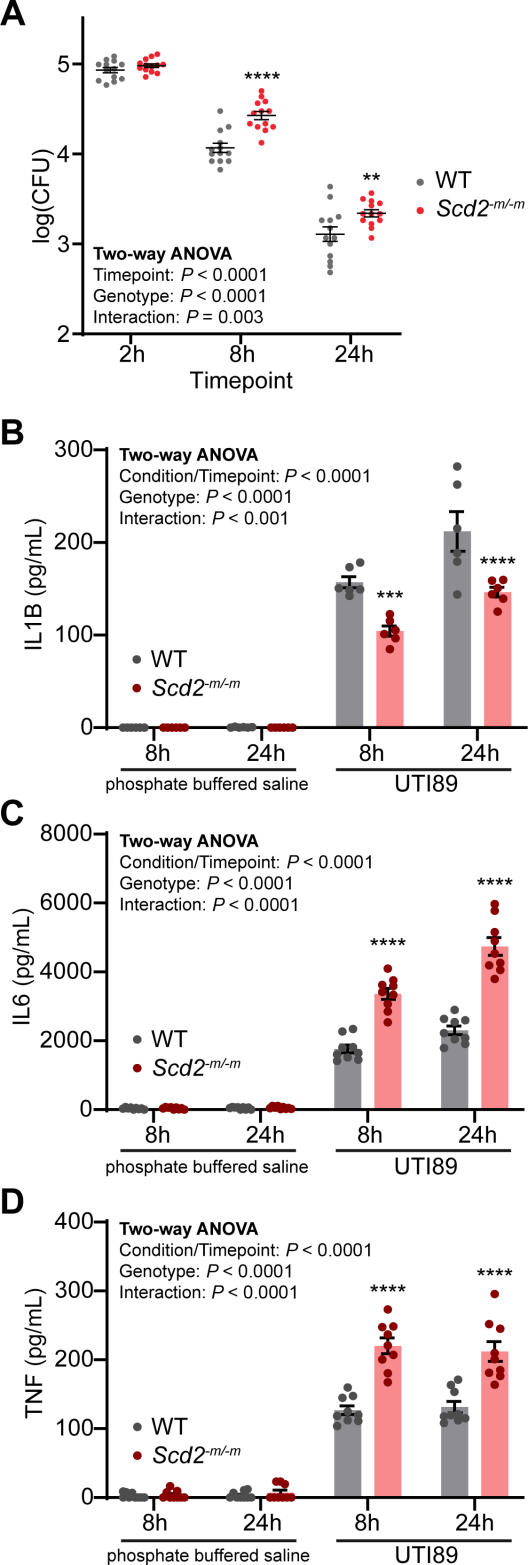
Deficiency of SCD2 perturbs macrophage response to uropathogenic *E. coli* infection. (**A**) Dot plot showing the intracellular bacterial load in *Scd2^-m/-m^* macrophages challenged with the uropathogenic *E. coli* strain UTI89 (multiplicity of infection [MOI] = 1). Each line indicates the mean ± SEM for *n* = 13 samples from three independent experiments. Statistical significance was assessed with two-way repeated measures (RM) analysis of variance (ANOVA) with matching on both factors and assuming sphericity, followed by a Šídák’s multiple comparisons test. (**B–D**) Bar graphs showing quantifications of IL1B, IL6, and TNF released by *Scd2^-m/-m^* macrophages challenged with the uropathogenic *E. coli* strain UTI89 (MOI = 1). Each bar indicates the mean ± SEM of *n* = 6–9 samples/group. Statistical significance was assessed using two-way ANOVA with Šídák’s multiple comparisons test. Statistical significance is indicated with asterisks as follows: **, *P* < 0.01; ***, *P* < 0.001; ****, *P* < 0.001. WT, wild type.

We have found that in macrophages, SCD2 deletion leads to dysfunction in inflammatory gene expression and response to LPS. In order to test the relevance of these impairments during the response to pathogens, we infected macrophages *in vitro* with the UTI89 strain of uropathogenic *E. coli*. We first assessed for intracellular bacterial burden in macrophages at 2, 8, and 24 h post-infection. We found that there was no difference in intracellular bacterial burden at 2 h post-infection, indicating that uptake or entry of bacteria into macrophages is not affected by SCD2 deficiency (Fig. 6A). However, at 8 and 24 h post-infection, there was significant increase in intracellular bacteria, indicating that when *Scd2* is deleted from macrophages, there is impaired clearance of intracellular *E. coli* (Fig. 6A).

Because there was dysregulation of inflammatory gene expression response to LPS (Fig. 1D and E), we measured IL1B, IL6, and TNF released by *Scd2^-m/-m^* macrophages challenged with UTI89. Consistent with impaired IL1B activation after LPS stimulation (Fig. 4A, B, G and H), *Scd2^-m/-m^* macrophages challenged with UTI89 released significantly less IL1B to the extracellular space (Fig. 6B). However, there was more IL6 and TNF released (Fig. 6C and D). Taken together, these data indicate that loss of *Scd2* from macrophages perturbs their response to UPEC infection.

## DISCUSSION

In this study, we delineate the cellular effects when *Scd2* is deleted from murine macrophages. First, we found that there were perturbations to inflammatory gene expression and response, providing independent validation of previous reports that SCD-deficient macrophages exhibit dysregulated inflammatory gene expression (7, 9, 10). We also found that although SCD2 was dispensable for general NFkB activation, loss of SCD2 impaired the activation of IL1B in response to inflammatory stimuli. We specifically identified that SCD2 is uniquely required for signal 1 activation of *Il1b* transcription and translation into precursor IL1B protein. As such, when SCD2 is absent, activation and release of IL1B are blunted upon macrophage stimulation with LPS or challenge with uropathogenic *E. coli*. Furthermore, we found that macrophage SCD2 deficiency led to disruption of autophagy with accumulation of intracellular autophagosomes. This finding is not surprising given previous reports that SCD deficiency in pancreatic β cells impairs autophagosome fusion with lysosomes (20). In macrophages, autophagic flux and inflammation are inversely related; thus, disruptions to autophagic clearance in *Scd2^-m/-m^* macrophages could lead to downstream activation of inflammatory gene expression such as those we found for *Il6, Tnf*, and *Ccl2*.

When we assessed for changes in lipids in *Scd2^-m/-m^* macrophages, we found that there were no changes in either of SCD2’s two substrates, FFA(16:0) and FFA(18:0). In addition, the products of SCD2 catalysis, FFA(16:1) and FFA(18:1), were either unchanged or mildly increased 1.4-fold, respectively. Though we found there was no transcriptional compensation by other *Scd* isoforms, there could still be post-transcriptional changes that compensate for loss of *Scd2*, which is the most highly expressed isoform in murine macrophages (9). An alternative hypothesis is that when biosynthesis of monounsaturated fatty acids stalls due to loss of SCD2, utilization of monounsaturated FFA by some downstream pathways is restricted. For example, we found that loss of SCD2 dramatically depletes unsaturated CL, which are primarily localized to the mitochondrial inner membrane. This de-prioritization of CL synthesis may enable maintenance of steady-state pools of unsaturated FFA along with phospholipids that are major components of the plasma membrane whose composition and organization critically regulate macrophage function (21). Of pertinence to the deficient priming of IL1B in *Scd2^-m/-m^* macrophages, previous studies have established a direct role for CL in the activation of the NLRP3 inflammasome that activates and releases IL1B (18, 19). Our data suggest that CL, particularly those containing unsaturated fatty acids, may also play important role in the production of pro-IL1B following LPS priming.

These findings provide a link between fatty acid desaturation by *Scd2* and fundamental macrophage effector functions. We previously reported that macrophage *Scd2* expression declines in aging and that this could promote pathologic ocular angiogenesis (7). However, age-related loss of macrophage SCD2 could be of potential significance to other pathologies of aging. For example, urinary tract infections (UTIs) are common bacterial infections that affect more than 50% of women in their lifetimes and are most often caused by UPEC. The burden of this disease is exacerbated in elderly women with over 10% reporting having a UTI within the past year (22) such that this infection is the leading cause of bacteremia in the elderly (23). This is further highlighted by UTI recurrence that cannot be cured by antibiotic therapy, which is seen in over 50% of elderly patients (24). In fact, women aged 55–64 suffer from the highest prevalence of recurrent UTIs (i.e., ≥3 UTIs/year) (25). Although reduced estrogen (26) and incontinence contribute to increased susceptibility, there may be other risk factors involved such as age-related dysfunction in immune response (3, 27). In this study, we tested the relevance of macrophage SCD2, whose expression declines in aging, in macrophage response to the uropathogenic *E. coli* strain UTI89. SCD2-deficient macrophages were less able to clear internalized uropathogenic *E. coli*, and there was impaired IL1B release but increased IL6 and TNF release. Therefore, SCD-mediated fatty acid desaturation could be a key molecular program whose downregulation in aging could perturb macrophage effector functions such as those essential for the immune response to UPEC (28), thus contributing to increased risk for recurrent UTIs in elderly women. If found to be relevant to human disease, SCD-mediated fatty acid desaturation could be a key metabolic step that maintains macrophage inflammatory responses important for many diverse pathologies of aging.

## METHODS

### Mice

We obtained mice with floxed *Scd2* alleles (*Scd2^f/f^)* (29, 30) from Hide Tsukamoto (Keck School of Medicine of the University of Southern California). We crossed these mice with mice carrying the lysozyme M-Cre transgene (31) to generate mice with myelomonocyte-specific deletion of *Scd2* (*Scd2^-m/-m^*). We used male and female mice aged 2–5 months old for experiments. The Institutional Animal Care and Use Committee of Washington University in St. Louis approved all animal experiments.

### Bone marrow-derived macrophages

To isolate bone marrow cells, we sacrificed mice by CO_2_ euthanasia, dissected out both tibias and femurs, and passed 8 mL of ice-cold Dulbecco's Modified Eagle Medium (DMEM) through each bone’s medullary cavity. We filtered the bone marrow aspirates through a 40-µm nylon mesh strainer, pelleted the cells, and re-suspended the pellet in macrophage differentiation media consisting of DMEM with 10% fetal bovine serum (FBS), 10% media conditioned in CMG cells (32), 1% penicillin/streptomycin, and 1% L-glutamine. We replenished differentiation media 3 days after initial plating and re-plated macrophages for experimentation 5–6 days after initial plating. After differentiation, we used mature macrophage media consisting of DMEM with 10% FBS, 2% CMG-conditioned media, 1% penicillin/streptomycin, and 1% L-glutamine.

### Gene expression studies

#### RNA isolation

We washed cells twice with phosphate buffered saline (PBS), then used TRIzol (Thermo Fisher Scientific, Waltham, Massachusetts, USA) and RNeasy Plus Mini Kit (Qiagen, Hilden, Germany) to extract total RNA following the manufacturer’s recommended protocol.

#### RNA sequencing and analysis

We quantified the quality and quantity of the RNA samples with an Agilent Bioanalyzer or 4200 Tapestation. All RNA samples (*n* = 6) were of high quality with RNA integrity numbers ≥9.3. We enriched our samples for mRNA by performing polyA selection according to the manufacturer’s recommended protocol. Then, we prepared sequencing libraries with standard protocols and sequenced the samples on an Illumina NovaSeq 6000 at the McDonnell Genome Institute at Washington University School of Medicine in St. Louis. RNA sequencing reads were aligned to the mouse genome (Ensembl release 76 primary assembly) with Spliced Transcripts Alignment to a reference (33). Finally, we performed standard analysis of gene-level features using EdgeR and Limma (34, 35). To identify dysregulation of pathways, we performed Gene Set Enrichment Analysis (11, 12).

#### Quantitative polymerase chain reaction (qPCR)

We reverse transcribed total RNA to cDNA using the High-Capacity cDNA Reverse Transcription Kit (Applied Biosystems, Waltham, Massachusetts, USA). Target gene expression was normalized to geometric mean of the following housekeeping genes: *18S, Actb, Gapdh,* and *Hprt*. We calculated gene expression differences using the ΔΔCt method.

### Enzyme-linked immunosorbent assay

To measure secreted cytokines, we collected media from BMDMs and measured mouse IL6, IL1B, or TNF using Quantikine Colorimetric Sandwich ELISA Kits (R&D Systems, Minneapolis, Minnesota, USA) following the manufacturer’s instructions.

### Immunocytochemistry

We plated cells in chamber slides. After incubation with any cell culture reagents, we washed cells with pre-warmed PBS and fixed cells in 4% paraformaldehyde or 10% neutral-buffered formalin for 15 min at room temperature. Then, we blocked and permeabilized cells with PBS containing 5% serum from the secondary antibody host and 0.3% Triton X-100. We incubated cells with primary antibody overnight at 4°C. We performed secondary antibody staining for 1 h at room temperature. The following primary antibodies were used: anti-P65 (8242 from Cell Signaling Technology, Danvers, Massachusetts, USA), anti-phosphorylated P65 (Cell Signaling Technology 3033), and anti-P62 (GP62-C from Progen, Heidelberg, Germany). We imaged cells using a Zeiss LSM800 confocal microscope.

### Immunoblotting

We loaded cell lysate containing ~15 µg protein into individual lanes, separated proteins using SDS-polyacrylamide gel electrophoresis, and then transferred proteins to a nitrocellulose membrane (0.22 µm pore size). We blocked membranes with 5% nonfat dry milk or bovine serum albumin (BSA) in PBS. We incubated membranes with primary antibodies overnight at 4°C, followed by secondary antibodies for 1 h at room temperature. We used the following primary antibodies: anti-P65 (Cell Signaling Technology 8242), anti-phosphorylated P65 (Cell Signaling Technology 3033), anti-IL1B (ab9722 from Abcam, Cambridge, United Kingdom), anti-caspase 1 (Cell Signaling Technology 83383), anti-gasdermin D (Cell Signaling Technology 39754), anti-P62 (Progen GP62-C), and anti-beta actin (A5316 from Sigma, St. Louis, Missouri, USA). We visualized blots using the dual-channel Odyssey CLx Imaging System and quantified protein bands of interest using Image Studio. We normalized signal for proteins of interest against β-actin as a loading control.

### Transmission electron microscopy

We fixed macrophages with 2% paraformaldehyde and 2.5% glutaraldehyde in 100 mM sodium cacodylate buffer (pH 7.2) for 1 h at room temperature. We washed samples with sodium cacodylate buffer and performed a post-fix in 1% osmium tetroxide for 1 h. We then rinsed samples with distilled water prior to *en bloc* staining with 1% aqueous uranyl acetate for 1 h. Following several rinses in distilled water, we dehydrated samples in a graded series of ethanol and embedded samples in Eponate 12 resin. We cut 95 nm sections with a Leica Ultracut UCT ultramicrotome, stained them with uranyl acetate and lead citrate, and imaged them on a JEOL 1200 EX transmission electron microscope equipped with an AMT 8.0 megapixel digital camera and AMT Image Capture Engine V602 software.

### Lipidomics

We assessed by liquid chromatography-mass spectrometry total cellular abundances of PC, PA, PE, PS, PI, PG, CL, TAG, FFA, carnitine, AC, and ACoA. We precipitated protein to extract PC, PA, PE, PS, PI, PA, PG, carnitine, AC, and ACoA. We extracted CL, FFA, and TAG using the Blyth-Dyer method. We added internal standards to samples prior to extraction. These were PC(14:1–14:1), PA(14:0–14:0), PE(16:1–16:1), PS(14:0–14:0), PI(16:0–16:0), PG(15:0–15:0), CL(14:0–14:0–14:0–14:0), TAG(17:0–17:0–17:0), d4-FFA(16:0), d9-carnitine, d3-AC(16:0), and d4-ACoA(16:0) for PC, PA, PE, PS, PI, PG, CL, TAG, FFA, carnitine, and short-chain AC (AC(2:0) and AC(3:0)), AC, and ACoA, respectively.

We analyzed PC, PE, PS, PI, PG, TAG, carnitine, and AC with a Shimadzu 20A HPLC system coupled to an API4000 mass spectrometer operated in positive multiple reaction monitoring (MRM) mode. We analyzed PA and CL in positive and negative MRM modes, respectively, on 4000QTRAP mass spectrometer coupled to a Shimadzu 20A HPLC system. We analyzed ACoA with a Shimadzu 20A HPLC system coupled to a 6500QTRAP + mass spectrometer operated in positive MRM mode. We processed data using Analyst 1.6.3.

To monitor instrument performance, we created a quality control sample by pooling aliquots of the study samples. This quality control sample was injected between every 10 samples. We report only the lipid species with coefficient variance less than 15% in the quality control sample. We calculated the ratio of analytes to their corresponding internal standards and reported relative quantifications comparing ratios in wild-type samples to those in *Scd2^-m/-m^* samples.

To assess for statistical significance comparing wild-type (*n* = 3) and *Scd2^-m/-m^* (*n* = 3) relative quantifications, we performed hypothesis testing using bootstrapping. Briefly, we constructed a null population (*n* = 99,999) of the differences between two sample means (*n* = 3 each). Both samples were created by random sampling with replacement from the pooled data. Finally, we calculated a two-sided *P*-value based on the percentile of the actual difference compared to the null population.

### Infection of BMDMs with UTI89

We infected BMDMs with the uropathogenic *E. coli* strain UTI89 for 2 h and measured intracellular CFU at 2, 8, and 24 h after the start of infection (15). We grew UTI89 statically in Luria-Bertani broth for 17 h at 37°C. We challenged BMDMs with UTI89 at an MOI of 1.0 for 2 h. After the 2-h infection period, we removed UTI89-containing media, washed BMDMs with PBS, and applied gentamicin to kill extracellular bacteria. We applied gentamicin at 100 µg/mL for either 15 min (for the 2-h timepoint) or 1 h (for the 8- and 24-h timepoints); after which, we switched to a lower 10 µg/mL gentamicin dose. After gentamicin treatment, we prepared cell lysates and then plated serial dilutions for CFU analysis.

### Statistics

We describe the statistical analyses used for bulk RNA sequencing and lipidomics in prior sections. For all other data, we performed statistical analyses using GraphPad Prism 9. We first assessed the normality of our data graphically and by using a Kolmogorov-Smirnov test. When comparing a single variable between two different groups, we used two-tailed *t* tests or two-tailed Mann-Whitney tests. For comparisons of a single variable among >2 groups, we used one-way or two-way analysis of variance with Šídák’s multiple comparisons test. A *P*-value <0.05 was considered statistically significant.

## Data Availability

Genomic data have been deposited in the Gene Expression Omnibus (GSE226259).
